# Supplementary material for: Compendium of medically important snakes, venom activity and clinical presentations in Ghana
Source: PLoS Negl Trop Dis. 2023 Jul 28;17(7):e0011050. doi: 10.1371/journal.pntd.0011050 (PMC10411737; doi:10.1371/journal.pntd.0011050)
Supplement: Attachment — Submitted filename: Response to Reviewers.doc [file pntd.0011050.s002.doc]

| Reviewer No. | Comment | Response |
| --- | --- | --- |
| REVIEWER NUMBER 1 | | |
| 1 | In many instances, I had the thought that authors have not properly proofread the text (see below for some examples): repeated information in tables, different formatting along the text, scientific names not written in italics, and so on.  I strongly recommend that they proofread the text. | Many thanks for this observation. We endeavoured to address each of these comments. Repeated information in tables and different formatting within the entire manuscript have all been addressed. We also corrected all scientific names as suggested. We have also proofread the entire manuscript to correct all typos and grammar issues. |
| 2 | Authors claim that their study seek to fill gaps about knowledge on venomous snakes. This is important in the context of snakebites in underdeveloped and rural areas like Ghana, but one important thing that is missing are pictures of the species assessed.  Both professionals and people living in areas with high risk of snakebites should be able to identify species of venomous snakes living in their area. I believe that those pictures would improve the manuscript and help the readers not familiar with local snakes.  A good source for general information may be found in: Chippaux & Jackson. 2019. Snakes of Central and Western Africa. | Thank you for this important suggestion. We have now included as an Appendix 1 the photos of the medically-important snakes mentioned in the manuscript (see Appendix 1). Thank you. |
| 3 | Were the authors able to retrieve information on which species that were the most common causes of snakebites (that is, which are medically more important), and which are the most common manifestations in their bites? If so, a section on results focusing on them is highly recommended | Data on snakebite is scanty at the medical facilities in Ghana, as such details on type of snakes involved in many cases are not included in any database for snakebites. This major gap is the topic of our global research. We however, acknowledge that should this data be extracted from the grey literature that would improve the manuscript. Data on snake bites do not include information such as type of snake, site of bite, nature of activity at the time of bite, time of day, incidence of bites in relation to the time of the year and whether anti-snake venom was given (Mensah et al., 2016).  Ref. Mensah EK, Karikari K, Aikins M, Vanotoo L, Sackey S, Ohuabunwo C, Wurapa F, Sifah TK, Afari E. Secondary analysis of snake bite data in the Western Region of Ghana: 2006-2010. Ghana medical journal. 2016 Jul 18; 50(2):103-6. |
| 4 | The main reference used in the tables of venom activities was a site that, as far as I could see, was updated last time in 2015. It would be very relevant and advisable that the authors add more recent references on the topics addressed along the text. | We used the University of Adelaide Clinical Toxinology Resources database which is a very comprehensive database for toxins and venoms. This database has not been updated since 2015 however, we acknowledge this suggestion and have provided other additional relevant references where appropriate. |
| 5 | Some sections are written in italics (e.g., 3.2.1.), some are not (e.g., 3.2.2.), and some are written in both ways (3.1.1.). Standardize. | This has been corrected throughout the manuscript. |
| 6 | Many scientific names are not in italics (e.g., names in tables) and some family names are not written in capital letter or misspelled. | These are errors that occurred as a results of formatting challenges during submission. These have now been corrected throughout the manuscript. All scientific names are written correctly (See Table 3, Table 4 etc). We thank you very much for these observations. |
| 7 | Figures and tables are not numbered in order (Figure 1, Figure 2, etc.). Please, fix that. | Figures and table have been numbered in their correct order in L150 (Figure 1); L172 (Figure 2); L 205 (Figure 3) and Lines 495 and 516 (Figure 4). Originally the figures were numbered according to the sectioning of the manuscript which we recognise will mislead readers. Thank you. |
| 8 | I have some issues with Figure 3.1.1., although I think this figure is absolutely necessary to the manuscript: some symbols representing the snakes in given map are too similar. I suggest authors to change them in order to a better view by the readers. In figure caption, authors say that it characterizes the habits of the snakes. I did not see that information, only on species names, range and type of environment. I am in doubt if the locations of the symbols are the actual distribution of snakes in the country, or only are representative of the environments that they are found.  I also would like to know which is the source for the distribution of the snakes. I leave a reference for distribution (maps included), in case of need: <https://www.gbif.org/> | Map has been revised as suggested and the symbology changed on page 14 of the revised manuscript.  The figure caption has been revised in Line 205 and now reads ‘”Figure 3: Characterisation of medically-important snakes in Ghana by name, distribution patterns and WHO categorization. Data sourced from grey literature and additionally data adapted from Global biodiversity information facility (<https://www.gbif.org/)>”. The symbols are just representations of environments those snakes have been reported to have occurred.  The snake distribution maps were constructed using information from grey literature and the habitat type and agro-ecological zones they were reported to have occurred in literature. We have additionally included <https://www.gbif.org/> |
| 9 | In some tables, there is a symbol representing one given information, and in others the information is written in the table. Please standardize. | This has been corrected in Table 1 |
| 10 | Lines 40 and 43: “Viperidae” instead of “viperidae".  All text: When speaking about snakes or snakebites, please use “venomous” instead of “poisonous”. Snakes are venomous (they produce venom and inject it through a specialized delivery system). Poisonous animals do not have this delivery system (e.g., toads, some fish).  Line 82 and 85: If incidence rates are given in number of inhabitants, please indicate clearly. | Lines 40-43: “Viperidae” has been used instead of “viperidae".  Throughout the manuscript ‘’venomous’’ has been used to replace “poisonous” as suggested.    Line 82: Snakebite incidence rates have been edited to reflect the number of inhabitants and now reads *“……56.4/100,000 and mortality at 1.35/100,000 inhabitants”*  Line 85: *“…580 per 100,000 and mortality of 16 per 100,000 inhabitants per annum’’* |
| 11 | Table 3.1.1. Which group of vine snakes are the authors referring to? African Thelotornis or others?  Lines 277 and 278: Did the authors mean “and are terrestrial in nature except the boomslang” instead of “and are terrestrial in nature except the Puff adder (Bitis arietans)”? Puff adder is terrestrial in nature, while boomslang is arboreal (which the authors themselves state on lines 262-624).  Line 277: The sentence “These species are mostly found in either savannah or rainforest and forest fringes and are terrestrial in nature except the Puff adder (*Bitis arietans*) has a nocturnal habit…” seems odd. Did the authors meant that puff adder is nocturnal or there is an excerpt of the sentence missing? | Yes, we meant the forest vine snake (*Thelotornis kirtlandii*). This has been clarified in Table 1 in the revised manuscript.  Lines 277 and 278: We meant ……. “and are terrestrial in nature except the boomslang *Dispholidus typus*”. These has been corrected in the revised manuscript in Line 266.  We acknowledge this comment and have clarified the statement to read “*These species are mostly found in either savannah or rainforest and forest fringes and are terrestrial in nature (except the boomslang Dispholidus typus which is arboreal). Species in this category belong to both WHO Cat 1 and 2 making them highly lethal (Figure 3 & Table 3.2.3)”* in line 277-279. |
| 12 | Table 3.2.3.: Please, indicate in the Table title that the species with procoagulant venoms are inserted in this table.  Table 3.2.5.: This table looks different from the others in the manuscript. The same is true for Table 3.3.1.  The terms “anti-venom”, “vaccines”, and “antiserum”, “anti-snake venoms” are used. Which one is the correct? Please, standardize.  Line 323: The reference style here is different from the style used in the rest of the text.  Table 3.3.1.: Can the authors provide references for information in that table?  In the same table, the antivenom SII Polyvalent Antisnake (Snake Antivenom Seum IP) (#9 in the table) does not list species from Africa. Is it really used for treatment in that continent?  Section 4: “elapids” instead of “elapidaes”. | Table 3.2.3 is now Table 4 in the revised manuscript and in the Table caption the statement …”the species with procoagulant venoms are inserted in this table” in Line 285 page 22.  Table 3.2.5 and 3.3.1 are now Table 6 and 7 and they present different information with details not the same as the earlier ones.  We have revised the use of these terms in the entire manuscript. We now only use the term snake antivenom unless it is a brand name. However, we believe where each is used is appropriate because these words are used interchangeably in the medical literature and is acceptable.  This has been corrected in the revised manuscript.  Information in Table 7 was retrieved from the registered product database of Food and Drug Administration as well as host of other literature. This has been cited.  This is available in Ghana and other parts of Africa. The purpose of Table 7 is to highlight the Anti-snake venom sera availability in Ghana.  Line 394: “elapidaes’’ is a typo and has been corrected to read ‘’elapids’’ |
| REVIEWER NUMBER 2 | | |
| 13 | There is need to clarify how the information from literature databases was reviewed. Was it systematically reviewed? If so, it would be advisable to follow the PRISMA guidelines. | Literature database was reviewed by themes using key words including: snakes, medical importance, snake bites, venom and venom type, envenomation, symptoms and signs, vaccines, venom expenditure, strike behaviour and venom-metering + Ghana, West Africa, Africa, World. Relevant literature containing these key words were compiled in matrix form alongside source data for validation. Those that addressed the research questions were then selected and relevant information extracted for use. This was not a typical systematic review but can be described as “thematic desk review”. |
| 14 | It would be helpful to provide more information on the sources of the data and how it was collected. For example, it is not clear how the data from the DHIMS database was augmented or what types of medical records were reviewed | The primary data is excluded from this updated review thus data sources said to have been used in augmenting the DHIMS and gray literature are no longer relevant. |
| 15 | It would be helpful to provide more information on the sampling method used for the study, including the criteria for selecting the personnel to be interviewed, the hospitals and health facilities to be included. | Primary data has been excluded since the secondary data had enough information to answer the research questions. |
| 16 | The study would need ethical clearance from an IRB | We have applied for IRB ethical clearance and awaiting feedback. However, with the exclusion of the primary data, ethical clearance is not necessarily needed. |
| 17 | This sentence is not clear  "Whereas a cardiotoxin, sarafotoxin, with endothelin-like properties has been isolated from the Variable Burrowing Asp"  "Bites from these species present as coagulopathy and lead to infarction and ischemia." - It might be clearer to say "Bites from these species can cause coagulopathy, infarction, and ischemia | Comment is well noted.  The statement has been revised as “*A cardiotoxin, sarafotoxin, with endothelin-like properties has been isolated from the Variable Burrowing Asp but, the cardiotoxin associated with West African Gaboon Viper is not yet characterized*”  This sentence has been revised accordingly. |
| 18 | The conclusions is too broad and focus the main findings or implications of the study more. The authors need to be more concise and with the discussion points. | The authors have reviewed the comment and feel the conclusions address the research questions posited and supported by the findings elicited. |
| 19 | The limitations of the analysis and study have not been included in this paper. | Comment noted with appreciation.  However, limitations were captured in the narrative though not as a specific sub-section.  We have accordingly inserted a limitations of study section as follows:  ***Limitations of study****:*  *Data from the District Health Information Management System (DHIMS) of the Ghana Health Service (GHS) is summarised information thus lack specific characteristics of snakes and snake bites in Ghana. Furthermore, application of conclusions from this paper must be used with circumspection in view of impact of climate change on snake distribution and atypical presentations of some snake venoms.(Line 506)* |
| 20 | The overall paper may also need minor grammar checks | Well noted. We have revised the manuscript to be free of minor errors. |
| 21 | One strength of the manuscript is the inclusion of data from multiple sources, including published literature and a government health database. The review is also specific to the context of Ghana, which is useful for understanding the local epidemiology of snake bite envenoming. | Comment well appreciated. |
| 22 | One potential weakness of the manuscript is that it is based on a desk review rather than original research.  It is also not clear whether the sample size for the data from the District Health Information Management System is sufficient to accurately represent the incidence of snake bite envenoming in Ghana. | Comment well noted.  The intention is to provide base information on snakes and snake bites in Ghana, expose the gaps in knowledge, paucity of the data (DHIMS) and propose and conduct research to fill out these gaps.  The DHIMS data base is an accumulation of all health events occurring in the Ghana Health System and does not require the computation of sample size.  Primary data is not included in this review. |
| 23 | The methodology is generally not very clear.  Overall, the manuscript appears to provide useful information on snake bite envenoming in Ghana and could be valuable for clinicians and researchers working in the region. However, further original research may be necessary to fully understand the epidemiology of snake bite envenoming in Ghana. | The study aimed at analyzing existing data to provide baseline data for future build-on. As such original research is not required at this stage of the overall multi-component project that this current paper is part of in our estimation. |
| 24 | Additional comments for the authors. Other specific issues  Introduction section:   1. The study objectives is not clearly stated in the introduction. The lines 114 to 116 should be clearer and connect with materiel in the previous paragraph.   In the methods section:   1. The criteria for selecting hospitals and regional medical stores is not described. 2. The interviews with the experts will also need a standardised questionnaire. 3. In the paragraph describing the data management process, it is not clear what is meant by "the matrix." It might be helpful to provide more context or explanation for this. 4. It would be helpful to provide more information on the sources of the data and how it was collected. For example, it is not clear how the data from the DHIMS database was augmented or what types of medical records were reviewed. 5. It would be helpful to provide more information on the sampling method used for the study, including the criteria for selecting the personnel to be interviewed, the hospitals and health facilities to be included. 6. The interview with the experts will also need ethical clearance form an institutional review board. 7. It might be helpful to provide more information on the study's limitations and any potential sources of bias or confounding factors (overall limitations of the study and details can also be written after the discussion).   In the results section:   1. It would be important to know what aspects the experts interviewed contributed to the results.   In the discussion section:   1. The link between climate change and human-snake encounters is not well-established and seems like it has been misplaced here. It would be appropriate if the first paragraph focuses on the study findings. 2. Generally, the discussion could be more concise and focused on the main findings and implications of the study. 3. There is some instances of repetition of information from the results section which can be trimmed to make the discussion more focused. 4. It will also be important to highlight what aspects where provided by experts and how they fit in the discussion. 5. A section focusing on the limitations of the research, methodology should be written   In the conclusion section:   1. The conclusion is too broad and does not clearly summarize the main findings or implications of the study. 2. A separate section focusing on recommendations can be written 3. The recommendation for building capacity for African pharmaceutical companies to produce antivenoms is not well-supported or explained. | Noted and reviewed  This has been excluded in this review. Data from the DHIMS and gray literature was sufficient in addressing our research questions.  This has also been excluded in this update and review.  The matrix describes a table of the themes as indicated in the text in the row and information source in the columns. Information extracted from the various sources are placed under the provided themes for validation.  In this review, the authors excluded data from the primary sources including medical records.  The authors thus worked with only the DHIMS data and gray literature using bibliographic software EndNote and search engine Google Scholar.  The primary data (including personnel to be interviewed, the hospitals and health facilities) has been excluded in this review and update thus sampling is no longer an issue. Data from the DHIMS and grey literature has been found sufficient in addressing the research questions.  With the exclusion of primary data including interviewing experts, selection of hospitals and facilities, ethical clearance is not necessarily required again. Nonetheless, the authors have submitted the proposal to the institutional review board for consideration and awaiting feedback in the coming days.  This is well noted.  Some limitations are provided in the narrative and a sub-section is being created to address this comment.  No potential source of bias has been observed in this review.  This section of the manuscript and research has been excluded. Data from the DHIMS and gray literature was sufficient to answer the research questions.  Well noted.  However, this is a preamble highlighting the importance of snake bites as a sequelae to climate change needing increasing attention.  This is well noted and the manuscript has been reviewed accordingly.  The tables were developed along the lines of expressed venoms and therefore with some snakes expressing more than one type of venom, it may reveal as a repetition.  This part of the manuscript has been excluded in this revised manuscript.  This is well noted. The limitations of the study have been captured in the revised manuscript.  This is well noted. The authors tried to provide summarized essential information in the algorithm (Figure 5) with a broad preceding narrative as a guide. It provides a quick reference of summarized information gathered in this review for the reader in a single picture.  Well noted and thank you. This is captured in section 7, line 512 to 522.  This is well noted. Details on this is provided in Table 7 in the results. The narrative on this in the discussion has been truncated to reduce duplication and verbosity. |
| 25 | Major revisions:   1. For the literature search - design a correct systematic review and report using PRISMA guidelines 2. For the expert survey - inclusion and exclusion criteria, sample size determination, standardised questionnaire | Literature search was done using themes relevant to the research questions in a “*structured thematic review*”. This was not a typical systematic review and the PRISMA guidelines may not necessarily apply.  This part of the manuscript has been excluded. |
| REVIEWER NUMBER 3 | | |
| 26 | The manuscript is well written, however with some minor grammatical errors and needs thorough correction before it is accepted. | All grammatical errors have been corrected in the revised manuscript. |
| 27 | Minor comments  Line 154: 2.2. Methods, it is a repetition of line 124 (2. Methods)  Line 212; 3.2.1. Snakes species and neurotoxin in humans  This is misleading, change as neurotoxic symptoms in human  238 3.2.2. Snake species and Cardiotoxins in humans.  Change as cardiotoxicity or cardiotoxic effect in human  Table 3.2.1: Snakes species and neurotoxin (Postsynaptic) effects in humans. Change as neurotoxic  238 3.2.2. Snake species and Cardiotoxins in humans  Change as cardiotoxic effect  251 Table 3.2.2: Snakes species and Cardiotoxin effects in humans  Change as cardiotoxic  256 3.2.3. Snake species and haemorrhagins (or haematotoxins) in humans  Change as hemorrhagic effect  272 3.2.4. Snake species and procoagulants in humans  Change as procoagulant effect  289 3.2.5. Snake species and cytotoxic and broad-spectrum toxins in humans  Change as cytotoxic effect and broad-spectrum toxicity. | Line 154:2.2. has been revised to “Data Search Strategy”  Line 212; 3.2.1 has been revised to read “Snake species with neurotoxic effects in humans”. Similar instances have all been revised including cardiotoxic effects (Line 238; 3.2.2; Line 248; Table 3.2.2), haemorrhagic/haematotoxic effects (Line 253) procoagulant effect (Line 270) and cytotoxic and broad-spectrum toxicity (Line 287) |
| 28 | Discussion  Line 349, Snakes that express neurotoxins are the most prevalent across Ghana though. Change this as neurotoxic venom.  Similarly change other properties all throughout the text.  Viz. cardiotoxicity or cardiotoxic effect (do not present as cardiotoxin or myotoxin or neurotoxin)  Lines 91 and 92 : *Compounding these challenges is the proliferation of poor quality antivenoms available in these areas.*  The current discussion justifying the above statement is inadequate, therefore throw little more light on this statement in the discussion. | Line 349, neurotoxins has been replaced with neurotoxic venom  All other instances have been modified  The discussion has been revised to cater for this comment. We have inserted the following sentences into the discussion in this regard. “That challenge however will be ensuring that these snake venom antisera meet the required quality standards especially for liquid formulated antisera which requires strict maintenance of the cold-chain throughout storage period; a situation that can be challenging in limited-resource settings. It would therefore require effective post marketing surveillance to provide the needed quality assurance while also ensuring that the cold-chain is maintained throughout the shelf life of these liquid snake venom antisera.” |
| 29 | Important issue:  The authors have given elaborate details on snakebite incidence and hot spots, clinical characteristics of snake venoms, the distribution pattern of medically important snakes of Ghana, snake species that induce various toxic properties such as neurotoxicity, myotoxicity, etc., available anti-venoms/anti-serum, however, the manuscript is seriously lacking about the information on mortality and morbidity details. Having acquired detailed data on the above-mentioned parameters, I believe, adding the mortality and morbidity details would elevate the quality of the manuscript. I suggest the authors, if possible, seriously consider the inclusion of the suggestion. | While we would have loved to include this report in our paper, we unfortunately do not have data on morbidity and mortality in the database that we reported from. It is however our strong recommendation that such data be captured in subsequent reporting in the DHIMS. This has been included in the revised manuscript |
