# Supplementary material for: Compendium of medically important snakes, venom activity and clinical presentations in Ghana
Source: PLoS Negl Trop Dis. 2023 Jul 28;17(7):e0011050. doi: 10.1371/journal.pntd.0011050 (PMC10411737; doi:10.1371/journal.pntd.0011050)
Supplement: Attachment — Submitted filename: Reviewer comments PNTD-D-22-01600.docx [file pntd.0011050.s001.docx]

Reviewer comments on manuscript **PNTD-D-22-01600**

This is an interesting work, that would be useful to health professional in Ghana and neighboring countries, regarding a neglected disease in a rural and underdeveloped area. However, I see some issues that I judge necessary to solve before this manuscript is ready to publication.

In many instances, I had the thought that authors have not properly proofread the text (see below for some examples): repeated information in tables, different formatting along the text, scientific names not written in italics, and so on. I strongly recommend that they proofread the text.

Authors claim that their study seek to fill gaps about knowledge on venomous snakes. This is important in the context of snakebites in underdeveloped and rural areas like Ghana, but one important thing that is missing are pictures of the species assessed. Both professionals and people living in areas with high risk of snakebites should be able to identify species of venomous snakes living in their area. I believe that those pictures would improve the manuscript and help the readers not familiar with local snakes. A good source for general information may be found in: Chippaux & Jackson. 2019. Snakes of Central and Western Africa.

Were the authors able to retrieve information on which species that were the most common causes of snakebites (that is, which are medically more important), and which are the most common manifestations in their bites? If so, a section on results focusing on them is highly recommended.

The main reference used in the tables of venom activities was a site that, as far as I could see, was updated last time in 2015. It would be very relevant and advisable that the authors add more recent references on the topics addressed along the text.

Some sections are written in italics (e.g., 3.2.1.), some are not (e.g., 3.2.2.), and some are written in both ways (3.1.1.). Standardize.

Many scientific names are not in italics (e.g., names in tables) and some family names are not written in capital letter or misspelled.

Figures and tables are not numbered in order (Figure 1, Figure 2, etc.). Please, fix that.

I have some issues with Figure 3.1.1., although I think this figure is absolutely necessary to the manuscript: some symbols representing the snakes in given map are too similar. I suggest authors to change them in order to a better view by the readers. In figure caption, authors say that it characterizes the habits of the snakes. I did not see that information, only on species names, range and type of environment. I am in doubt if the locations of the symbols are the actual distribution of snakes in the country, or only are representative of the environments that they are found. I also would like to know which is the source for the distribution of the snakes. I leave a reference for distribution (maps included), in case of need: <https://www.gbif.org/>

In some tables, there is a symbol representing one given information, and in others the information is written in the table. Please standardize.

Lines 40 and 43: “Viperidae” instead of “viperidae".

All text: When speaking about snakes or snakebites, please use “venomous” instead of “poisonous”. Snakes are venomous (they produce venom and inject it through a specialized delivery system). Poisonous animals do not have this delivery system (e.g., toads, some fish).

Line 82 and 85: If incidence rates are given in number of inhabitants, please indicate clearly.

Table 3.1.1. Which group of vine snakes are the authors referring to? African *Thelotornis* or others?

Lines 277 and 278: Did the authors mean “and are terrestrial in nature except the boomslang” instead of “and are terrestrial in nature except the Puff adder *(Bitis arietans)*”? Puff adder is terrestrial in nature, while boomslang is arboreal (which the authors themselves state on lines 262-624).

Line 277: The sentence “These species are mostly found in either savannah or rainforest and forest fringes and are terrestrial in nature except the Puff adder *(Bitis arietans)* has a nocturnal habit…” seems odd. Did the authors meant that puff adder is nocturnal or there is an excerpt of the sentence missing?

Table 3.2.3.: Please, indicate in the Table title that the species with procoagulant venoms are inserted in this table.

Table 3.2.5.: This table looks different from the others in the manuscript. The same is true for Table 3.3.1.

The terms “anti-venom”, “vaccines”, and “antiserum”, “anti-snake venoms” are used. Which one is the correct? Please, standardize.

Line 323: The reference style here is different from the style used in the rest of the text.

Table 3.3.1.: Can the authors provide references for information in that table?

In the same table, the antivenom SII Polyvalent Antisnake (Snake Antivenom Seum IP) (#9 in the table) does not list species from Africa. Is it really used for treatment in that continent?

Section 4: “elapids” instead of “elapidaes”.
